# Modeling the oxygen uptake kinetics during exercise testing of patients with chronic obstructive pulmonary diseases using nonlinear mixed models

**DOI:** 10.1186/s12874-016-0173-8

**Published:** 2016-06-01

**Authors:** Florent Baty, Christian Ritz, Arnoldus van Gestel, Martin Brutsche, Daniel Gerhard

**Affiliations:** Department of Pulmonary Medicine, Cantonal Hospital St. Gallen, Rorschacherstrasse 95, St. Gallen, 9007 Switzerland; Department of Nutrition, Exercise and Sports, University of Copenhagen, Rolighedsvej 26, Frederiksberg C, 1958 Denmark; School of Mathematics & Statistics, University of Canterbury, New Zealand, Private Bag 4800, Christchurch, 8140 New Zealand

**Keywords:** Nonlinear mixed effects, Modeling, Chronic obstructive pulmonary disease, Exercise testing, Oxygen kinetics

## Abstract

**Background:**

The six-minute walk test (6MWT) is commonly used to quantify exercise capacity in patients with several cardio-pulmonary diseases. Oxygen uptake ($\dot {\mathrm {V}}$O_2_) kinetics during 6MWT typically follow 3 distinct phases (rest, exercise, recovery) that can be modeled by nonlinear regression. Simultaneous modeling of multiple kinetics requires nonlinear mixed models methodology. To the best of our knowledge, no such curve-fitting approach has been used to analyze multiple $\dot {\mathrm {V}}$O_2_ kinetics in both research and clinical practice so far.

**Methods:**

In the present study, we describe functionality of the **R** package medrc that extends the framework of the commonly used packages drc and nlme and allows fitting nonlinear mixed effects models for automated nonlinear regression modeling. The methodology was applied to a data set including 6MWT $\dot {\mathrm {V}}$O_2_ kinetics from 61 patients with chronic obstructive pulmonary disease (disease severity stage II to IV). The mixed effects approach was compared to a traditional curve-by-curve approach.

**Results:**

A six-parameter nonlinear regression model was jointly fitted to the set of $\dot {\mathrm {V}}$O_2_ kinetics. Significant differences between disease stages were found regarding steady state $\dot {\mathrm {V}}$O_2_ during exercise, $\dot {\mathrm {V}}$O_2_ level after recovery and $\dot {\mathrm {V}}$O_2_ inflection point in the recovery phase. Estimates obtained by the mixed effects approach showed standard errors that were consistently lower as compared to the curve-by-curve approach.

**Conclusions:**

Hereby we demonstrate the novelty and usefulness of this methodology in the context of physiological exercise testing.

**Electronic supplementary material:**

The online version of this article (doi:10.1186/s12874-016-0173-8) contains supplementary material, which is available to authorized users.

## Background

The 6-Minute Walk Test (6MWT) is routinely used to quantify submaximal exercise capacity in patients with chronic cardio-pulmonary diseases [1]. It is considered as the test of choice to quantify functional capacity and patient’s daily life activities [2]. Portable wireless cardiopulmonary exercise testing devices enable to measure breath-by-breath oxygen exchange kinetics during exercise performance. From the original acquired breath-by-breath oxygen exchange data, curves can be generated and parameters can be estimated by curve model fitting [3, 4]. These parameters provide information about the interaction of the cardiovascular-, cardiac autonomic-, pulmonary-, and metabolic system.

In the field of pulmonary medicine, there is a growing interest in estimating exercise parameters capable of objectively evaluating the functional capacity of patients with chronic obstructive pulmonary disease (COPD). Time course measurements of oxygen consumption ($\dot {\mathrm {V}}$O_2_) provide key physiological determinants of exercise capacity which are hardly influenced by conditions other than the underlying disease. Modeling $\dot {\mathrm {V}}$O_2_ kinetics during exercise testing in patients with COPD is therefore a revelant topic.

Traditionally, individual $\dot {\mathrm {V}}$O_2_ uptake curves have been analyzed visually and parameters representing for example the time needed for a 50 % increase in oxygen uptake (T _1/2_) were quantified in an inconsistent manner [5]. Nowadays, $\dot {\mathrm {V}}$O_2_ kinetics can be accurately modeled by fitting nonlinear regression models [6–8]. A modeling approach insures that parameters are estimated in an objective and systematic manner, fully exploiting the information available in the kinetics data.

Several difficulties arise when dealing with nonlinear regression models. Unlike linear regression where mathematical solutions exist for the estimation of the best fitted parameters, nonlinear regression often requires additional information from users who should specify among others, the equation of the model, a set of starting parameters for the minimization procedure, and pay a particular attention to the check of the models assumptions [8].

Routinely acquired oxygen uptake kinetics generate batches of curves whose parameters are generally estimated using curve-by-curve model fits. The oxygen kinetics are sometimes originated from individuals which can be grouped into categories (e.g., distinct diagnostic group, disease severity, etc.) and one might be interested in testing differences between parameter estimates with regard to these pre-defined categories. Hypothesis testing is classically done in two integral steps. First, curve fitting is used and as a result a set of parameter estimates can be produced. In a second step, inference based on the estimated parameters is carried out. However, in this type of data, within-group correlation typically arise and specific regression techniques must be used [9]. Mixed models are well-suited for the analysis of grouped data. They allow to explicitely incorporate intra-group correlations by means of random effects and get joint estimates of the regression model parameters [10]. Fitting nonlinear mixed models comes with the same type of challenges as fitting nonlinear regression models.

The whole analytical workflow requires various statistical expertise. The aim of the current work is to demonstrate how recent methodological developments enable to carry out these series of successive procedures — namely the simultaneaous fit of nonlinear models within the mixed effects framework, followed by inferences based on parameter estimates — in an automated and all-integrated manner, by using recently developed packages from the **R** statistical software [11].

The next sections are organized as follows. The novel mixed effects methodology is described in the “Material and methods”. A detailed example of physiological exercise testing from patients with COPD is provided in the “Results”. The mixed models approach is compared with a traditional curve-by-curve approach. The statistical and physiological relevance of the findings is exposed in the “Discussion”.

## Material and methods

### COPD dataset

Patients with COPD referred to the Department of Pulmonary Medicine of the University Hospital of Basel (Switzerland) for a 6MWT gave their informed consent to participate to the study. The data analyzed in the present study were fully anonymized and no individual clinical data are presented. The study was approved from the local institutional review board (Ethikkommission beider Basel). The study was conducted in accordance with the principles enunciated in the Declaration of Helsinki and the guidelines of Good Clinical Practice. Further details on the study can be found on previous publications [7, 12, 13]. The supporting data set is provided in Additional file 1.

### Oxygen monitoring during 6MWT

We used the Oxycon Mobile ^*Ⓡ*^ (Viasys Healthcare, USA) portable, wireless cardiopulmonary exercise testing device to measure breath-by-breath $\dot {\mathrm {V}}$O_2_ kinetics. Pulse rate was determined by using an ECG-triggered belt (Polar ^*Ⓡ*^ Electro OY T-61). Blood oxgen saturation level (SpO_2_) was measured by using a finger clip. $\dot {\mathrm {V}}$O_2_ and carbon dioxide output ($\dot {\mathrm {V}}$CO_2_), tidal volumes and breathing frequency were assessed by using a facemask (dead space <70 mL) with a flow sensor and a gas analyzer. The patient carried data storage and transfer units by using a dedicated harness. Wireless transfer of breath-by-breath data to a laptop computer allowed real-time monitoring. The additional weight (950 g) of the equipment has no effect on walking distance [12]. The exact 6MWT procedure with mobile telemetry has been previously described [12].

Original breath-by-breath data were imported from the mobile telemetry device. Raw data were pre-processed by averaging the breath-by-breath measurements over consecutive periods of 20 s.

### Oxygen uptake kinetics during exercise testing

**Fig. 1:**
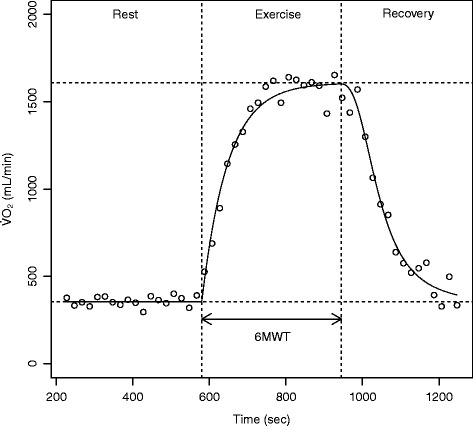
Oxygen kinetics before, during and after the 6MWT exercise testing

The American Thoracic Society (ATS) published detailed instructions regarding how to conduct the 6MWT as much standardized as possible. Accordingly, the 6MWT consists of 3 phases; five minutes of rest, six minutes of walking and five minutes of recovery. The oxygen uptake kinetics ($\dot {\mathrm {V}}$O_2_) of each phase can be modeled by nonlinear regression as demonstrated in Fig. 1. A constant O_2_ consumption is expected in the resting phase, followed by a 6-minute monotonic O_2_ increase, and a progressive recovery phase where O_2_ decreases back to its initial level.

Established parameters for the assessment of cardiopulmonary exercise capacity in patients with COPD during the 6MWT are the oxygen uptake at steady state ($\dot {\mathrm {V}}\mathrm {O}_{2}ss$) and the six-minute walking distance (6MWD) [14].

The incline of $\dot {\mathrm {V}}$O_2_ during the initial phase of low-intensity exercise ($\dot {\mathrm {V}}$O_2_ on-kinetics) provides important information about oxygen delivery and muscle metabolism, and are found to be noticeably delayed in patients with several chronic cardiac- and pulmonary diseases [15–19]. $\dot {\mathrm {V}}$O_2_ on-kinetics can be quantified by the time (mean response time: MRT) required for $\dot {\mathrm {V}}$O_2_ to achieve 63 % of the $\dot {\mathrm {V}}\mathrm {O}_{2}ss$ in response to exercise [20, 21].

Patients with COPD are limited during exercise by dyspnea and fatigue and also have difficulties to recover normal breathing after exercise. As the limited cardio-pulmonary reserve in patients with COPD appears to affect exercise responses it may be postulated that it also affects the recovery phase. Recovery kinetics of oxygen uptake ($\dot {\mathrm {V}}$O_2_ off-transient kinetics) reflect the ability to recover from exercise that is indicative of daily life.

$\dot {\mathrm {V}}$O_2_ off-transient kinetics can be quantified by both the steepness of the curve as well as the time necessary for $\dot {\mathrm {V}}$O_2_ to recover by 50 % from its peak effort value ($\mathrm {T}_{1/2}\dot {\mathrm {V}}\mathrm {O}_{2}$).

### An interpretable nonlinear regression model

A six-parameter nonlinear regression model describing the 3 main phases of the oxygen kinetics (before, during and after 6MWT) is defined as follows:

1 $$ \dot{\mathrm{V}}\mathrm{O}_{2}(t) = \left\{ \begin{array}{ll} \text{if}~ t \le \lambda: & \dot{\mathrm{V}}\mathrm{O}_{2}rest,\\ \text{if}~ \lambda < t \le \lambda + 360: & \dot{\mathrm{V}}\mathrm{O}_{2}rest + (\dot{\mathrm{V}}\mathrm{O}_{2}ss - \dot{\mathrm{V}}\mathrm{O}_{2}rest) (1 - e^{-(t-\lambda) / \tau_{1}}),\\ \text{if}~ t > \lambda + 360: & \dot{\mathrm{V}}\mathrm{O}_{2}rest + (\dot{\mathrm{V}}\mathrm{O}_{2}ss - \dot{\mathrm{V}}\mathrm{O}_{2}rest) (1 - e^{-(t-\lambda) / \tau_{1}}) + \\ & (\dot{\mathrm{V}}\mathrm{O}_{2}recovery - \dot{\mathrm{V}}\mathrm{O}_{2}ss) / (1 + \exp(\tau_{2} * log((t - (\lambda_{max} + 360)) / \mathrm{T}_{1/2}\dot{\mathrm{V}}\mathrm{O}_{2}))) \end{array}\right.  $$

with $\dot {\mathrm {V}}\mathrm {O}_{2}rest$, $\dot {\mathrm {V}}\mathrm {O}_{2}ss$ and $\dot {\mathrm {V}}\mathrm {O}_{2}recovery$ the oxygen level at rest, steady state during exercise and recovery, respectively; *τ*_1_ the growth rate of the mono-exponential $\dot {\mathrm {V}}$O_2_ function during 6MWT; *τ*_2_ the steepness of the exponential decay during the recovery phase and $\mathrm {T}_{1/2}\dot {\mathrm {V}}\mathrm {O}_{2}$ the time for half decrease of the $\dot {\mathrm {V}}$O_2_ level in the recovery phase. All the 6 previously mentioned parameters ($\dot {\mathrm {V}}\mathrm {O}_{2}rest$, $\dot {\mathrm {V}}\mathrm {O}_{2}ss$, *τ*_1_, $\mathrm {T}_{1/2}\dot {\mathrm {V}}\mathrm {O}_{2}$, *τ*_2_, $\dot {\mathrm {V}}\mathrm {O}_{2}recovery$) have to be estimated by nonlinear regression procedure. *λ* is the length of the resting period. It is controlled by the experimenter who decides when the resting period ends (usually after 5 min of rest) by initiating the 6MWT. The experimenter reports manually the exact duration of the resting period (*λ*) which is therefore not estimated during the fitting procedure. *λ*_*max*_ corresponds to the length of the longest resting period in the set of kinetics. This value is known a priori hence not estimated during the fitting procedure. It is simply used to “align” multiple kinetics by removing differences among the duration of individual resting phases.

This nonlinear model takes into account the basic experimental and physiological specificities of a 6MWT, including a resting phase whose duration is controlled by the experimenter, followed by an immediate mono-exponential raise of oxygen during 6-min exercise, followed by a progressive decline of $\dot {\mathrm {V}}$O_2_ during the recovery phase. It is worth noting that this six-parameter model including a single mono-exponential function that describes the oxygen increase during exercise is a rather common choice [6] which suits well the purpose of our simple clinical application. More complex models describing the increase of oxygen consumption by means of two- or three-term exponential function have been proposed. However, they are only useful for the modeling of physiological processes occurring in specific situations such as heavy-intensity exercise [22, 23].

### Mixed effects modeling

Following the notation of Davidian and Giltinan [10], a nonlinear regression model for hierarchical data (e.g., patients and 6MWT measurements nested within patients) can be defined in two stages: First modeling the variability within the *i*^th^ patient, and hereby incorporating between-patient variation.

Stage 1: For *i*=1,⋯,*m* individuals, the following models can be assumed:

$$y_{ij} = \dot{\mathrm{V}}\mathrm{O}_{2}(t_{ij}, \beta_{i}) + \epsilon_{ij} $$

where *y*_*ij*_ are the response vectors of length *j*=1,⋯,*n*_*i*_ with the corresponding vectors individual times *t*_*ij*_. The nonlinear function such as the above six-parameter model (Eq. 1) evaluated at time *t*_*ij*_ is denoted by $\dot {\mathrm {V}}\mathrm {O}_{2}(t_{ij}, \beta _{i})$ with a *p*-dimensional individual-specific parameter *β*_*i*_. The residual vectors $\epsilon _{ij} \sim \mathcal {N}(0, \sigma ^{2} \Lambda _{i})$ are assumed to be normally distributed with a correlation structure defined by the elements of matrices *Λ*_*i*_; for the COPD data we will assume that *Λ*_*i*_ is the identity matrix.

The curve is described by the functions $\dot {\mathrm {V}}\mathrm {O}_{2}(t_{ij}, \beta _{i})$ with an individual-specific (*p*×1) vector of parameters *β*_*i*_.

Stage 2: Between-patient effects are described by modeling the *β*_*i*_. These effects are separated into fixed and random effects as in an ordinary linear mixed model (except there is no residual error as it was already introduced in Stage 1):

$$\beta_{i} = A_{i} \beta + B_{i} b_{i} $$

where *β* is the vector of fixed-effects parameters, which may differ between patients according to recorded patient characteristics encoded in the design matrix *A*_*i*_ (e.g., age or sex). Differences between patients, which are not captured by the recorded patient characteristics, are described by the patient-specific random effects vector *b*_*i*_; these random effects may possibly also be modified through explanatory variables encoded in the corresponding design matrix *B*_*i*_ (e.g., time).

As the random effects are intended for capturing inexplicable effects, which may balance out on average, they may be assumed to follow a mean-zero, possibly multivariate normal distribution: $b_{i} \sim \mathcal {N}(0, G)$ where *G* denotes the between-patient variance-covariance matrix, which we assumed to be entirely unstructured such all entries (covariance and variance parameters) were estimated from the data.

In our example, the overall mixed model was parametrized as follows: each individual $\dot {\mathrm {V}}$O_2_ kinetics defines one cluster for which a different fixed effect curve is assumed depending on the disease severity stage (all 6 parameters: $\dot {\mathrm {V}}\mathrm {O}_{2}rest$, $\dot {\mathrm {V}}\mathrm {O}_{2}ss$, *τ*_1_, $\mathrm {T}_{1/2}\dot {\mathrm {V}}\mathrm {O}_{2}$, *τ*_2_, $\dot {\mathrm {V}}\mathrm {O}_{2}recovery$); random effects were specified for the five parameters $\dot {\mathrm {V}}\mathrm {O}_{2}rest$, $\dot {\mathrm {V}}\mathrm {O}_{2}ss$, *τ*_1_, $\mathrm {T}_{1/2}\dot {\mathrm {V}}\mathrm {O}_{2}$, *τ*_2_. In this particular case, *A*_*i*_ is defined as the dummy coded design matrix specifying disease stage specific fixed-effect parameters, and *B*_*i*_ is defined as the random effect design matrix using dummy coded patient identifiers for 5 of the nonlinear model parameters.

We used Akaike’s information criterion (AIC) for identifying the appropriate random effects structure [24, 25].

### Sensitivity analysis

The curve-by-curve approach provides parameter estimates from fitting a nonlinear regression model (Eq. 1) to each individual patients kinetics data. Subsequently, for each of the six model parameters the corresponding parameter estimates of all patients may be analyzed by means of analysis of variance models to obtain estimates for each disease stage. This two-step curve-by-curve approach only relies on being able to fit the patient curves one by one. This approach resulted in estimated coefficients that could be interpreted as population level effects whereas nonlinear mixed effects models allow patient-specific interpretations.

### Implementation

The whole analytical workflow relies on the **R** packages drc [26] and nlme [27] for the nonlinear regression with mixed effects. The newly developed package medrc [28] elegantly combines functionalities of the packages drc and nlme. Finally the package multcomp was used for the statistical inference [29]. The main features of these packages are summarized below.

- drc allows simultaneous fit of several nonlinear regression models [26]. It provides automated fit of a list of nonlinear models by directly specifying initial parameter values (self-starters) for the estimation of the nonlinear model parameters. The main function in drc is *drm()*, which requires as arguments the name of the data set, the self-starter model, the dependent and indepedent variables.
- nlme provides tools for the fit of Gaussian nonlinear mixed models [27]. It supports specifying correlation structure for residuals and is particulary adapted for repeated measures designs. This package will be used indirectly through the package medrc described next.
- medrc combines the automated nonlinear regression modeling framework of the package drc with the nonlinear mixed estimation framework of the package nlme. The function *medrm()* in medrc allows to fit nonlinear mixed models, by providing the following arguments: *form* the formula with the response ($\dot {\mathrm {V}}$O_2_) on the left side as a function of the independent variable (time) on the right side; *curveid* the name of the categorical variable that divides the dataset into several clusters; *data* the name of the data set; *fct* the definition of the model; *random* the definition of the random effects. medrc is available at the following github repository: https://github.com/daniel-gerhard/medrc
- multcomp [29] is used for parameter inference, by providing functionalities for multiple tests for the fixed effects of the mixed models.

A sample **R** code is provided in Additional file 2.

## Results

### Application to six-minute walk test oxygen kinetics in patients with COPD

$\dot {\mathrm {V}}$O_2_ kinetics were measured in 61 patients with COPD who were traditionally classified into 3 disease severity stages (GOLD II, III and IV). A summary of the patient characteristics is presented in Table 1.

**Table 1:** Anthropometrics, pulmonary functions, cardio-pulmonary exercise capacity. Values are presented as median [IQR]

	COPD disease stage		
	II	III	IV
Anthropometrics			
Subjects, n	21	30	10
Female/male	10/11	10/20	5/5
Age, yr	72.0 [59.0–77.0]	67.5 [61.0–71.0]	60.5 [52–62]
BMI (kg/m^2^)	28.1 [25.5–32.0]	24.3 [21.8–28.0]	20.0 [18.8–20.6]
Pulmonary functions			
FEV_1_, L	1.6 [1.3–1.8]	1.0 [0.8–1.1]	0.7 [0.7–0.8]
FEV_1_, % predicted	59.0 [58.0–66.0]	36.5 [34.0–42.0]	26.5 [26.0–28.0]
FEV_1_/FVC, ratio	0.6 [0.5–0.6]	0.4 [0.3–0.5]	0.4 [0.3–0.4]
Exercise capacity			
6MWD, m	370.0 [300.0–438.0]	352.5 [290.0–392.0]	345.0 [265.0–374.0]

BMI: body mass index; FEV_1_: forced expiratory volume in 1 sec; FEV_1_/FVC ratio: forced expiratory volume in 1 sec (FEV_1_) expressed as percent of the forced vital capacity (FVC); 6MWD: 6-minute walking distance

The six-parameter nonlinear regression model was jointly fitted to the set of 61 oxygen kinetics using the function *medrm()* in the package medrc. The initial model selection of the random effects structure resulted in the minimum AIC for a model with random effects assigned to five out of the six model parameters; results from this model are reported below. The detailed results of the model selection process are provided in the Additional file 3: Table S1. It is worth noting that the model with random effects assigned to all six model parameters did not converge. Graphical check of the residuals is provided in the Additional file 4: Figure S1.

**Fig. 2:**
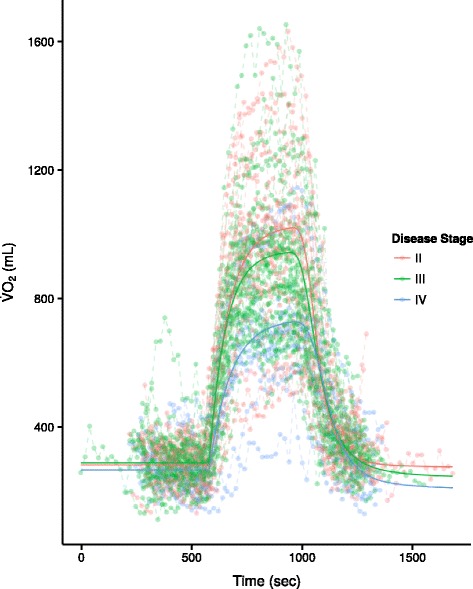
Oxygen kinetics and fitted curves summarized within each COPD disease stage. COPD disease stages II, III and IV are represented by *red*, *green* and *blue* lines respectively

Figure 2 displays the 61 raw oxygen kinetics data together with the fitted curves summarized within each COPD disease severity stage. The five estimated variance parameters for the random effects were: $SD_{\tau _{1}} = 44.09$; $SD_{\dot {\mathrm {V}}\mathrm {O}_{2}rest} = 58.26$; $SD_{\dot {\mathrm {V}}\mathrm {O}_{2}ss} = 250.51$; $SD_{\tau _{2}} = 1.05$; $SD_{\mathrm {T}_{1/2}\dot {\mathrm {V}}\mathrm {O}_{2}} = 44.31$.

Significant differences between disease stages were found regarding steady state oxygen uptake during exercise testing ($\dot {\mathrm {V}}\mathrm {O}_{2}ss$ II vs. IV, adj. *p*=0.038), oxygen level after recovery ($\dot {\mathrm {V}}\mathrm {O}_{2}recovery$ II vs. IV, adj. *p*=0.0013) and inflection point in the recovery phase ($\mathrm {T}_{1/2}\dot {\mathrm {V}}\mathrm {O}_{2}$ II vs. IV, adj. *p*=0.088). There were significant differences when comparing the peak oxygen level reached during exercise ($\dot {\mathrm {V}}\mathrm {O}_{2}ss$) between moderate and very severe COPD disease stages. On average, patients with COPD stage IV reached a $\dot {\mathrm {V}}\mathrm {O}_{2}ss$ 292.5 mL (*se*=96.8) lower than patients with stage II during the 6MWT. These cardio-pulmonary limitations are also reflected by the differences found in the recovery phase. Patients with stage IV needed on average 49.7 (18.1) additional seconds to reduce half of their oxygen consumption in the recovery phase as compared to patients with stage II.

**Fig. 3:**
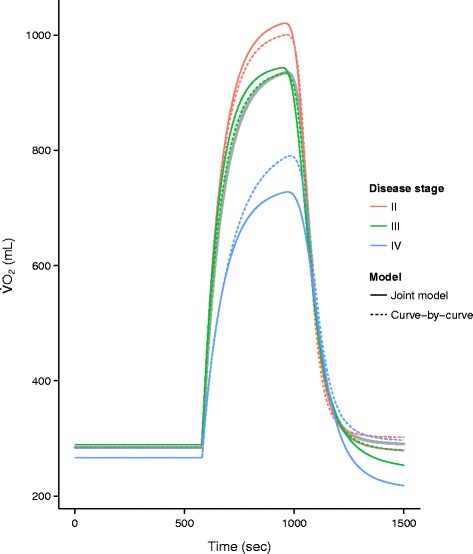
Comparison of the joint model with the curve-by-curve fits. The overall mean is represented by a thick *gray* line

Figure 3 shows in parallel the fitted curves obtained from the joint nonlinear mixed model and the curves obtained from the curve-by-curve approach. The overall mean curve obtained by pooling all data from all patients is represented by a thick gray curve. The estimates obtained from both approaches are given in Table 2, which shows that the standard errors are consistently (with one exception) smaller when using the mixed model (up to three times smaller).

**Table 2:** Comparison of the estimates provided by the joint mixed model approach and the curve-by-curve approach

Parameter	Disease stage	Joint mixed model		Curve-by-curve	
		Estimate	Std. error	Estimate	Std. error
*τ*_1_	II	84.15	10.14	82.42	18.15
	III	74.97	8.43	82.28	24.35
	IV	97.12	15.78	128.45	32.58
$\dot {\mathrm {V}}\mathrm {O}_{2}rest$	II	282.9	13.27	284.52	13.62
	III	288.82	11.09	284.04	18.27
	IV	266.79	19.25	283.08	24.44
$\dot {\mathrm {V}}\mathrm {O}_{2}ss$	II	1029.6	55.17	1007.7	58.46
	III	948.53	46.14	940.83	78.43
	IV	737.07	79.94	815.41	104.93
*τ*_2_	II	–3.77	0.28	–4.68	0.41
	III	–2.91	0.23	–3.74	0.54
$\dot {\mathrm {V}}\mathrm {O}_{2}recovery$	II	276.03	7.51	301.3	18.8
	III	242.53	9.8	276.03	25.22
	IV	209.71	15.03	294.65	33.75
T$_{1/2} \dot {\mathrm {V}}$O_2_	II	129.14	9.9	127.65	9.53
	III	134.7	8.46	136.51	12.79
	IV	178.82	15.27	161.6	17.11

*τ*_1_: growth rate of the mono-exponential $\dot {\mathrm {V}}$O_2_ function during 6MWT; $\dot {\mathrm {V}}\mathrm {O}_{2}rest$, $\dot {\mathrm {V}}\mathrm {O}_{2}ss$ and $\dot {\mathrm {V}}\mathrm {O}_{2}recovery$: oxygen level at rest, steady state during exercise and recovery, respectively; *τ*_2_: steepness of the exponential decay during the recovery phase; $\mathrm {T}_{1/2}\dot {\mathrm {V}}\mathrm {O}_{2}$: time for half decrease of the $\dot {\mathrm {V}}$O_2_ level in the recovery phase

## Discussion

A set of oxygen kinetics data that originated from a 6MWT in patients with COPD could be successfully analyzed both using a joint nonlinear mixed model and using a two-step curve-by-curve approach. Joint modeling is challenging when it comes to selecting the appropriate random effects structure, which is an essential part of the model as it allows to separate out patient variation, as models may not converge. We found that the more random effects were included the better the model as judged by AIC. However, for mixed models there exist alternative information criteria such as the conditional AIC [30] which perhaps may strike a better balance between model complexity and computational feasability. In contrast, the curve-by-curve approach is simpler, more robust, and operational. By averaging the patient specific curve-by-curve estimates, the possibly varying standard errors of the individual estimates are not taken into account. Specifically, comparison of results of the joint mixed model approach with the classical curve-by-curve approach revealed differences between the two approaches. The fitted regression curves obtained by the curve-by-curve approach were shrunk towards the overall population mean curve, demonstrating the well-known effect of attenuation on the marginal estimates as compared to conditional estimates [10].

However, the key difference between the two approaches was the reduction in estimated standard errors when using a mixed model approach, which successfully managed to remove a substantial part of the between-patient variation in the fixed-effects estimates. Our study indicates that the joint nonlinear mixed model is to be preferred over the two-step curve-by-curve approach. In general joint modeling should be preferred if the between-patient variation is large as we would expect it to be in many biological and medical studies [10]. Another advantage of joint modeling is that curves with few time points may still be fitted as estimation in the mixed model borrows strength from curves with many time points to curves with few time points. The curve-by-curve approach may fail to utilize such scarce data.

There is some evidence that misspecification of the distributions of the random effects (as normal distributions) may not severely impact inference on fixed effects parameters [31]. However, it is crucial to ensure approximately normally distributed residual errors. One approach is the transform-both-sides approach, which was originally proposed for ordinary nonlinear regression models, but it may be extended to nonlinear mixed-effect regression models: it was used with the Box-Cox family of power transformations, which include the logarithm [32]. A related approach is to model the residual variance in terms of a covariate [33]. An entirely different approach would be to consider models with other distributional assumptions than normality. To our knowledge such models are only readily available within a Bayesian framework (e.g., the MONOLIX software [34]) and still they involve considerable manual programming in specialized software.

## Conclusion

Recent statistical developments provide experimenters with a variety of tools for fitting nonlinear mixed models. Within the statistical environment **R** the package medrc provides a comprehensive and flexible framework for the parametrisation and inference of hierarchical nonlinear mixed-effects regression models with various biological and medical applications, as exemplified by an application from pulmonary medicine.

## Additional files

Additional file 1 Supporting oxygen kinetics data set. (RDA 151 kb)

Additional file 2 Sample R code. (R 388 kb)

Additional file 3 Model selection for the random effects structure. (PDF 661 kb)

Additional file 4 Graphical check of the residuals. (EPS 777 kb)
