# Supplementary material for: Modeling the oxygen uptake kinetics during exercise testing of patients with chronic obstructive pulmonary diseases using nonlinear mixed models
Source: BMC Med Res Methodol. 2016 Jun 1;16:66. doi: 10.1186/s12874-016-0173-8 (PMC4888741; doi:10.1186/s12874-016-0173-8)
Supplement: Additional file 3 — Model selection for the random effects structure. (PDF 661 kb) [file 12874_2016_173_MOESM3_ESM.pdf]

Table 1: Model selection for the random effects structure. The number reported in bold corresponds to the minimum AIC value.

| **Number of parameters** | **Set of parameters** | **AIC** |
|---|---|---|
| 6 | $\tau_1$, $\dot{V}O_2rest$, $\dot{V}O_2ss$, $\tau_2$, $\dot{V}O_2recovery$, $T_{1/2}\dot{V}O_2$ | No convergence |
| 5 | $\tau_1$, $\dot{V}O_2ss$, $\tau_2$, $\dot{V}O_2recovery$, $T_{1/2}\dot{V}O_2$ | 36138.24 |
| | $\tau_1$, $\dot{V}O_2rest$, $\tau_2$, $\dot{V}O_2recovery$, $T_{1/2}\dot{V}O_2$ | 37605.20 |
| | $\tau_1$, $\dot{V}O_2rest$, $\dot{V}O_2ss$, $\dot{V}O_2recovery$, $T_{1/2}\dot{V}O_2$ | 35627.84 |
| | $\tau_1$, $\dot{V}O_2rest$, $\dot{V}O_2ss$, $\tau_2$, $T_{1/2}\dot{V}O_2$ | **35562.27** |
| | $\tau_1$, $\dot{V}O_2rest$, $\dot{V}O_2ss$, $\tau_2$, $\dot{V}O_2recovery$ | No convergence |
| | $\dot{V}O_2rest$, $\dot{V}O_2ss$, $\tau_2$, $\dot{V}O_2recovery$, $T_{1/2}\dot{V}O_2$ | No convergence |
| 4 | $\tau_1$, $\dot{V}O_2rest$, $\dot{V}O_2ss$, $\tau_2$ | No convergence |
| | $\tau_1$, $\dot{V}O_2rest$, $\dot{V}O_2ss$, $\dot{V}O_2recovery$ | No convergence |
| | $\tau_1$, $\dot{V}O_2rest$, $\dot{V}O_2ss$, $T_{1/2}\dot{V}O_2$ | 35740.55 |
| | $\tau_1$, $\dot{V}O_2rest$, $\tau_2$, $\dot{V}O_2recovery$ | 37696.03 |
| | $\tau_1$, $\dot{V}O_2rest$, $\tau_2$, $T_{1/2}\dot{V}O_2$ | 37616.73 |
| | $\tau_1$, $\dot{V}O_2rest$, $\dot{V}O_2recovery$, $T_{1/2}\dot{V}O_2$ | No convergence |
| | $\tau_1$, $\dot{V}O_2ss$, $\tau_2$, $\dot{V}O_2recovery$ | No convergence |
| | $\tau_1$, $\dot{V}O_2ss$, $\tau_2$, $T_{1/2}\dot{V}O_2$ | No convergence |
| | $\tau_1$, $\dot{V}O_2ss$, $\dot{V}O_2recovery$, $T_{1/2}\dot{V}O_2$ | No convergence |
| | $\tau_1$, $\tau_2$, $\dot{V}O_2recovery$, $T_{1/2}\dot{V}O_2$ | No convergence |
| | $\dot{V}O_2rest$, $\dot{V}O_2ss$, $\tau_2$, $\dot{V}O_2recovery$ | No convergence |
| | $\dot{V}O_2rest$, $\dot{V}O_2ss$, $\tau_2$, $T_{1/2}\dot{V}O_2$ | 36108.11 |
| | $\dot{V}O_2rest$, $\dot{V}O_2ss$, $\dot{V}O_2recovery$, $T_{1/2}\dot{V}O_2$ | 36201.94 |
| | $\dot{V}O_2rest$, $\tau_2$, $\dot{V}O_2recovery$, $T_{1/2}\dot{V}O_2$ | No convergence |
| | $\dot{V}O_2ss$, $\tau_2$, $\dot{V}O_2recovery$, $T_{1/2}\dot{V}O_2$ | 36555.65 |
| 3 | $\tau_1$, $\dot{V}O_2rest$, $\dot{V}O_2ss$ | 36636.20 |
| | $\tau_1$, $\dot{V}O_2rest$, $\tau_2$ | 38113.85 |
| | $\tau_1$, $\dot{V}O_2rest$, $\dot{V}O_2recovery$ | 38105.69 |
| | $\tau_1$, $\dot{V}O_2rest$, $T_{1/2}\dot{V}O_2$ | 37859.51 |
| | $\tau_1$, $\dot{V}O_2ss$, $\tau_2$ | No convergence |
| | $\tau_1$, $\dot{V}O_2ss$, $\dot{V}O_2recovery$ | No convergence |
| | $\tau_1$, $\dot{V}O_2ss$, $T_{1/2}\dot{V}O_2$ | No convergence |
| | $\tau_1$, $\tau_2$, $\dot{V}O_2recovery$ | No convergence |
| | $\tau_1$, $\tau_2$, $T_{1/2}\dot{V}O_2$ | 37884.73 |
| | $\tau_1$, $\dot{V}O_2recovery$, $T_{1/2}\dot{V}O_2$ | No convergence |
| | $\dot{V}O_2rest$, $\dot{V}O_2ss$, $\tau_2$ | No convergence |
| | $\dot{V}O_2rest$, $\dot{V}O_2ss$, $\dot{V}O_2recovery$ | 36794.65 |
| | $\dot{V}O_2rest$, $\dot{V}O_2ss$, $T_{1/2}\dot{V}O_2$ | 36291.01 |
| | $\dot{V}O_2rest$, $\tau_2$, $\dot{V}O_2recovery$ | No convergence |
| | $\dot{V}O_2rest$, $\tau_2$, $T_{1/2}\dot{V}O_2$ | No convergence |
| | $\dot{V}O_2rest$, $\dot{V}O_2recovery$, $T_{1/2}\dot{V}O_2$ | No convergence |
| | $\dot{V}O_2ss$, $\tau_2$, $\dot{V}O_2recovery$ | No convergence |
| | $\dot{V}O_2ss$, $\tau_2$, $T_{1/2}\dot{V}O_2$ | 36604.57 |
| | $\dot{V}O_2ss$, $\dot{V}O_2recovery$, $T_{1/2}\dot{V}O_2$ | 36675.12 |
| | $\tau_2$, $\dot{V}O_2recovery$, $T_{1/2}\dot{V}O_2$ | No convergence |
| 2 | $\tau_1$, $\dot{V}O_2rest$ | 38462.77 |
| | $\tau_1$, $\dot{V}O_2ss$ | No convergence |
| | $\tau_1$, $\tau_2$ | 38359.71 |
| | $\tau_1$, $\dot{V}O_2recovery$ | No convergence |
| | $\tau_1$, $T_{1/2}\dot{V}O_2$ | No convergence |
| | $\dot{V}O_2rest$, $\dot{V}O_2ss$ | 37163.22 |
| | $\dot{V}O_2rest$, $\tau_2$ | No convergence |
| | $\dot{V}O_2rest$, $\dot{V}O_2recovery$ | No convergence |
| | $\dot{V}O_2rest$, $T_{1/2}\dot{V}O_2$ | No convergence |
| | $\dot{V}O_2ss$, $\tau_2$ | No convergence |
| | $\dot{V}O_2ss$, $\dot{V}O_2recovery$ | 37152.10 |
| | $\dot{V}O_2ss$, $T_{1/2}\dot{V}O_2$ | 36732.64 |
| | $\tau_2$, $\dot{V}O_2recovery$ | No convergence |
| | $\tau_2$, $T_{1/2}\dot{V}O_2$ | No convergence |
| | $\dot{V}O_2recovery$, $T_{1/2}\dot{V}O_2$ | 40375.75 |
| 1 | $\tau_1$ | 38688.74 |
| | $\dot{V}O_2rest$ | No convergence |
| | $\dot{V}O_2ss$ | 37452.69 |
| | $\tau_2$ | 40738.59 |
| | $\dot{V}O_2recovery$ | 40600.97 |
| | $T_{1/2}\dot{V}O_2$ | 40374.39 |

$\tau_1$: growth rate of the mono-exponential $\dot{V}O_2$ function during 6MWT; $\dot{V}O_2rest$, $\dot{V}O_2ss$ and $\dot{V}O_2recovery$: oxygen level at rest, steady state during exercise and recovery, respectively; $\tau_2$: steepness of the exponential decay during the recovery phase; $T_{1/2}\dot{V}O_2$: time for half decrease of the $\dot{V}O_2$ level in the recovery phase; AIC: Akaike Information Criterion.
